# Low-level of mitochondrial DNA alterations during direct-acting antiviral treatment in leukocytes of people who inject drugs living with HCV

**DOI:** 10.1128/spectrum.01946-26

**Published:** 2026-10-09

**Authors:** Laetitia Gay, Clara Rapenne, Valérie Desquiret-Dumas, Roselyne Vallo, Quynh Bach Thi Nhu, Linh Le Thi Thuy, Huong Thi Duong, Binh Nguyen Thanh, Catherine Quillet, Hong Thi Tran, Thanh Nham Thi Tuyet, Oanh Khuat Thi Hai, Vinh Vu Hai, Khue Pham Minh, Nicolas Nagot, Didier Laureillard, Pascal Reynier, Jean-Pierre Molès

**Affiliations:** 1 Pathogenesis and Control of Chronic and Emerging Infections, Montpellier University, INSERM, University of Antilles, CHU Montpellier 27038, Montpellier, France; 2 Department of Biochemistry and Molecular Biology, University Hospital of Angers 26995 , Angers, France; 3 University of Angers, MITOVASC Research Unit, CNRS 6015, INSERM U1083 26995, Angers, France; 4 HaiPhong Medical University 666972, Haiphong, Vietnam; 5 Supporting Community Development Initiatives, Hanoi, Vietnam; Shandong First Medical University, Jinan, Shandong, China

**Keywords:** direct-acting antivirals, drug users, genotoxicity, HCV treatment, mitochondria

## Abstract

Direct-acting antivirals (DAAs) have revolutionized hepatitis C virus (HCV) treatment, offering high cure rates and favorable safety profiles. However, concerns remain regarding their long-term mitochondrial safety, particularly among vulnerable populations such as people who inject drugs (PWID), who are frequently exposed to multiple mitotoxic agents. This longitudinal study investigated mitochondrial DNA (mtDNA) instability, including deletions and single-nucleotide variants, among PWID living with HCV during DAA treatment, using whole mitochondrial genome sequencing. We analyzed samples of 33 PWID living with HCV before treatment (baseline), at the end of treatment (week 12), and after long-term follow-up (week 48), in comparison with 30 HCV-seronegative PWID. Associations with clinical events were assessed during a 3-year follow-up after study completion. mtDNA deletions were detected in 9.1% of PWID living with HCV at baseline and increased to 19.2% post-treatment, whereas no mtDNA deletions were observed in HCV-seronegative PWID. Heteroplasmy levels (i.e., proportion of mutated to wild-type mtDNA) remained below 1%. Variants were widespread in both HCV-positive and HCV-seronegative PWID, predominantly in the non-coding D-loop region. A small number of low-heteroplasmy treatment-emergent variants were detected during follow-up, but none persisted at long-term follow-up. No significant associations were found between mtDNA instability and hepatic or splenic complications assessed at 3 years. These findings suggest that DAA exposure is associated with low-level mtDNA instability in blood leukocytes. Nonetheless, long-term mitochondrial surveillance is warranted, especially in post-mitotic tissues, to fully assess the implications of cumulative mitotoxic exposures in this high-risk population.

**IMPORTANCE:**

Direct-acting antivirals (DAAs) have revolutionized hepatitis C virus (HCV) treatment, but their safety is still under debate. Given the nature of these regimens, mitochondrial genotoxicity could represent a potential off-target effect. We searched for mitochondrial DNA alterations in people who inject drugs living with HCV. The prevalence of HCV infection is high in this population, and any potential toxicity would be exacerbated as they are exposed to other mitotoxic drugs. We detected low-level mitochondrial DNA alterations in blood leukocytes, including a modest increase in mitochondrial DNA deletions, but these alterations remained at very low levels (below 1%) and were not associated with any clinical complications during 3 years of follow-up. These findings provide reassuring evidence regarding the short-term mitochondrial safety of DAA treatment in blood leukocytes while supporting the need for longer-term studies in post-mitotic tissues.

**CLINICAL TRIALS:**

This study is registered with Clinical Trial Registry as NCT03526939 and NCT03537196.

## INTRODUCTION

Direct-acting antiviral (DAA) agents are now widely used to cure people living with hepatitis C virus (HCV), offering strong efficacy against all HCV genotypes and a good safety profile (1). This breakthrough has allowed the World Health Organization to set the goal of HCV elimination by 2030 (90% reduction in HCV incidence, 65% reduction in mortality, and 80% reduction in HCV-positive people treated) (2). However, attention remains about the long-term safety of DAAs. While acute side effects are rare, potential associations with hepatocellular carcinoma (HCC) occurrence and cardiovascular events have been debated (3–5). Given their structure, DAAs may induce mitochondrial DNA (mtDNA) genotoxicity (6–8). Indeed, nucleoside analogs, through off-target inhibition of host mitochondrial polymerases, can cause mtDNA depletion, impaired oxidative phosphorylation, and increased oxidative stress, consequently promoting mtDNA point mutations and/or deletions (9, 10). Sofosbuvir (SOF) and daclatasvir (DCV), for example, are known to induce mitochondrial stress in yeast (11). Unlike nuclear DNA polymerases, mtDNA polymerase γ has more limited proofreading (3′–5′ exonuclease) activity, making the mitochondrial genome more prone to genetic damage and instability (12).

A growing body of literature links mtDNA alterations to age-related diseases, such as neurodegenerative disorders and cancers, including HCC (13, 14). mtDNA instability has been well illustrated among people living with HIV, where mtDNA mutations accumulate during antiretroviral therapy (ART) and facilitate the occurrence of mitochondrial dysfunction (15–19). In tumors, mtDNA mutations arise at a rate comparable to those in the most common cancer driver genes and frequently affect specific subunits of the oxidative phosphorylation system (14). Whether these mutations represent true pathogenic events or rather an adaptive response to the new environment remains a conundrum. Recent genetic analyses suggest that specific mtDNA variants may predispose individuals to HCC development (20, 21). A deletion in the mtDNA encoded *ND6* gene has been shown to impair complex I stability and function in HCC tissue, illustrating a direct link with mitochondrial respiratory dysfunction (21).

Viral infections and treatments can damage mitochondria, affecting their mass, dynamics, and function, potentially contributing to liver disease progression (22). The HCV epidemic disproportionately affects people who inject drugs (PWID), a population exceeding 13.9 million worldwide, more than half of whom have been exposed to HCV (23). PWID also bear a high burden of HIV infection. This population is therefore particularly exposed to mitochondrial genotoxic drugs, including DAAs, ARVs, as well as other illicit drugs. In one of our studies, we identified that poly-exposure to DAAs and regular heroin or methamphetamine consumption were positively associated with mtDNA copy loss, while DAAs and ARV treatments were identified as risk factors for having the common age-related Δ4977 mtDNA deletion (24).

mtDNA damage may persist long after virus/treatment exposures (22). Studies have shown that mtDNA point mutations and large deletions remain prevalent among HIV-exposed but uninfected children years after the cessation of HIV and ARV drug exposure (25, 26). The consequences of these somatic mutations may have clinical consequences or not, according to their levels of pathogenicity and heteroplasmy (proportion of mutated to wild-type mtDNA). We also observed long-lasting mtDNA instability among HCV-cured PWID, with mtDNA deletions increasing even 9 months after DAA treatment discontinuation, while remaining subclinical (27).

Given that previous work did not comprehensively characterize mtDNA alterations across the mitochondrial genome, our primary objective was to screen for mtDNA alteration pattern of PWID during their exposure to HCV and DAA drugs using whole mitochondrial genome sequencing (WmtGS), to ascertain whether these alterations persist after discontinuation of these exposures, and to identify the associated risk factors.

## MATERIALS AND METHODS

### Study design

This study combined a cross-sectional observation of mtDNA alterations among PWID at baseline (before treatment initiation) and a longitudinal case series describing and analyzing the effects of DAA treatment. Time points were “before the treatment” (Baseline), “at the end of the treatment” (W12), and “at the end of the study” (W48). Associations between mtDNA alterations and clinical events were assessed during a 3-year follow-up after study completion.

### Study population

The present study was nested within the DRIVE (NCT03526939) and the DRIVE-C studies (NCT03537196) (28, 29). PWID were recruited during a respondent-driven sampling survey (RDSS) conducted by community workers in Hai Phong, Vietnam, in 2018. Active PWID were defined by a positive urine test result for heroin and recent injection marks. A total of 1444 PWID were enrolled in this RDSS (29). Out of them, HCV RNA-positive PWID were treated with combinations of sofosbuvir, daclatasvir, and/or ribavirin for 12 weeks. Among the participants who achieved successful treatment after 12 weeks and had available samples at baseline, W12, and W48, we selected 51 PWID for this study. Thirty-eight HCV-seronegative PWID were identified during the initial HCV screening phase as control population. Demographic, drug use, biological, and clinical data were obtained from the DRIVE and DRIVE-C database, which includes a clinical follow-up time point at 3 years after study completion.

### Sample collection and processing

PWID whole blood was collected at baseline, W12, and W48 on dried blood spots (DBS, Whatman 903 cards, GE Healthcare Bio-Sciences Corp., Chicago, IL, USA). DBS were stored in a zipped pouch with desiccant at −20°C. Then, DNA was extracted using the QIAamp DNA Blood Mini Kit (Qiagen, Hilden, Germany), following the manufacturer’s instructions, from 6 mm diameter punches (*n* = 3) and stored at −80°C.

### Detection and quantification of mitochondrial DNA instability

The mtDNA deletions and point mutations were assayed by experiments combining short-fragment whole mitochondrial genome sequencing and an in-house dedicated sequence analysis algorithm. Briefly, mtDNA was amplified in two overlapping fragments using PCR primers previously tested on Rho zero cells, which are devoid of mtDNA, in order to check the absence of amplification of mtDNA-related pseudogenes present in the nuclear genome. Library fragmentation and adapter ligation were generated by tagmentation using bead-linked transposomes (DNA Prep Kit, Illumina, San Diego, CA, USA). Bridged PCR and paired-end sequencing of 2 × 250 bp were performed on the MiSeq apparatus (MiSeq Reagent Kit v2, Illumina). The generated FASTQ files were processed in our in-house pipeline for coverage analysis, variant calling, annotation, and prioritization. Briefly, sequences were aligned to the mtDNA reference sequence (NC_012920.1) using BWA v0.7.17 tool, and variant calling was performed with Strelka v2.9.10 and MuTect v2.2.

mtDNA rearrangements were determined using eKLIPse v1.8, a diagnostic tool developed for the detection and quantification of large mtDNA rearrangements (30). The criteria to validate a deletion event were as follows: (i) a minimum sequencing depth of 100 reads per base, or (ii) a minimum number of 10 reads bearing the deletion event, or (iii) a heteroplasmy level above 0.05%.

**Fig 1:**
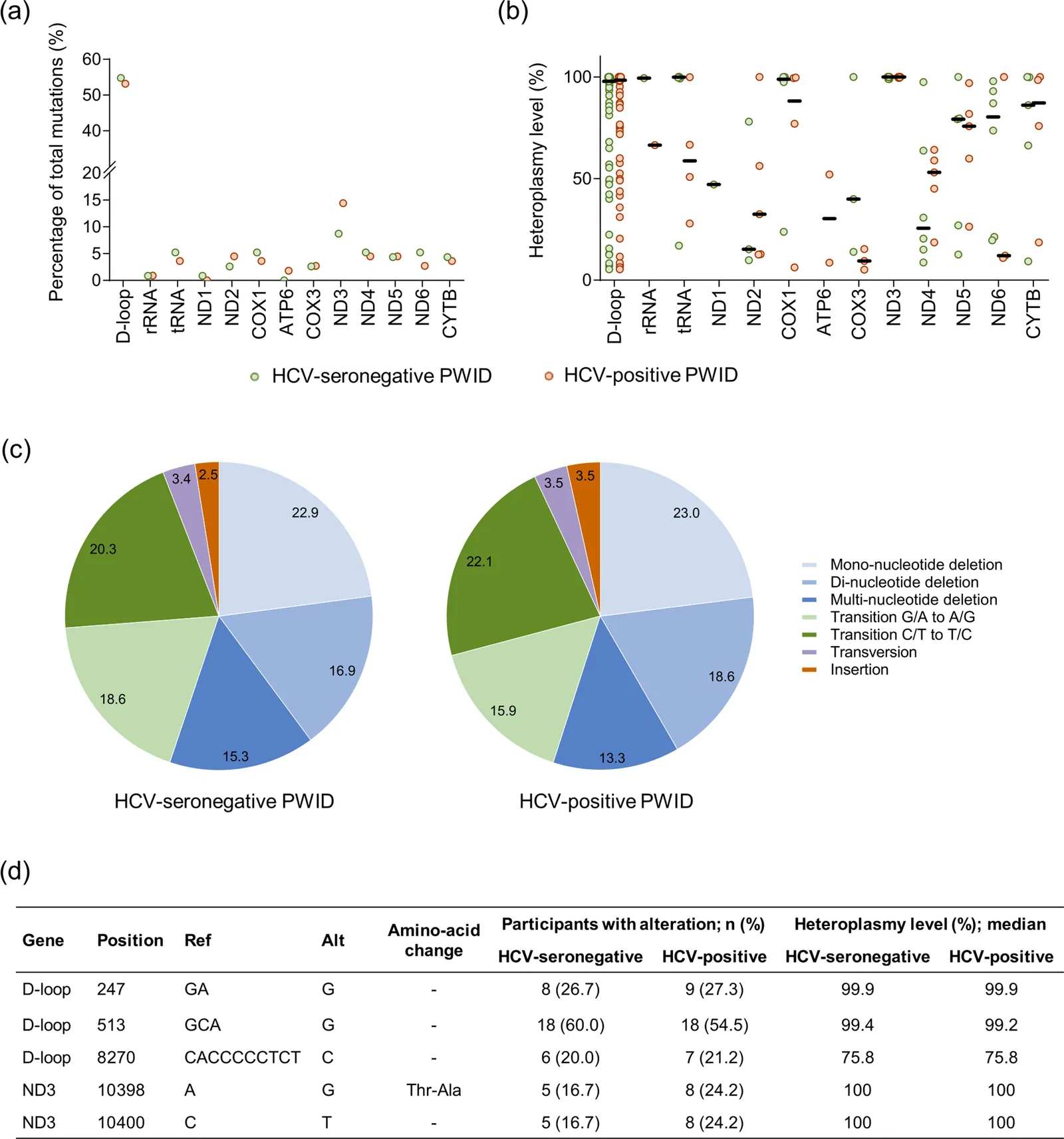
Comparison of mtDNA mutation profiles between HCV-positive and HCV-seronegative PWID. (**a**) Distribution of mtDNA variants and (**b**) heteroplasmy levels of mtDNA variants across mitochondrial genomic regions. (**c**) Distribution of variant types, expressed as percentages (*n* = 118 variants in HCV-seronegative PWID; *n* = 113 in HCV-positive PWID). (**d**) Descriptive table of the most frequently observed mtDNA variants (Ref, reference allele; Alt, alternative allele).

A variant filtering was performed on VCF files to prioritize the variants by excluding frequent sequencing artifacts and variants defining the patient haplogroup. The remaining variants were analyzed individually to determine biological relevance according to the following criteria: (i) status in the MITOMAP database, (ii) frequency in mtDNA variant databases (GenBank, gnomAD, and Helix), (iii) conservation percentage of the nucleotide, and (iv) classification of the variant by *in silico* pathogenicity predictor tools (MitImpact, APOGEE, SIFT, and PolyPhen). For the analysis, we focused on treatment-emergent variants, defined as those appearing at W12. We then assessed their persistence over time, i.e., variants detected at W12 that remained present at W48. In this analysis, variants that were frequently observed at baseline in the overall PWID population—namely those at positions 247, 513, 8270, 10398, and 10400 (Fig. 1d)—were not included, as they likely represent background variants rather than treatment-emergent changes.

### Statistical analysis

Participant characteristics are described using medians with interquartile range (IQR) for continuous variables and percentages for categorical variables. For the study of risk factors associated with mtDNA deletions, we performed a univariate analysis using Fisher’s exact test or Wilcoxon rank test to assess the association between having mtDNA deletions at baseline and the following variables: gender, age, HCV infection, HIV infection, receiving ARV treatment, and drug consumption. Heteroplasmy rate exceeding 1% is a threshold for suspicion of possible-related clinical outcomes. Associations between mtDNA deletions and clinical outcomes (the occurrence of hepatic or splenic complications: coarse liver, hepatomegaly, or splenomegaly) at long-term follow-up (3 years after study completion) were assessed using Firth’s penalized logistic regression models, in order to reduce potential bias due to the small sample size. Models were adjusted for participants’ baseline status for each outcome. We also assessed the change in liver stiffness (median liver stiffness measured by FibroScan) between baseline and long-term follow-up by calculating the difference for each patient. This change was then compared between participants with mtDNA alterations and those without using the non-parametric Wilcoxon rank-sum test. The threshold for statistical significance was set at *α* < 0.05. Statistical analyses were performed using Stata 16.1 (Copyright © 1985–2019, StataCorp LLC, College Station, Texas, USA).

## RESULTS

### Study population characteristics

Of the 51 PWID treated for HCV infection and 38 HCV-seronegative participants included in this study, DNA amplification and WmtGS sequences were successfully obtained for 33 PWID living with HCV and 30 HCV-seronegative participants. This suboptimal inclusion rate mainly resulted from the low quantity of intact mtDNA in DBS samples and the high sequencing coverage threshold required to validate the data set. The population of PWID living with HCV was predominantly men (90.9%) with a median age of 43 years. Participants received either SOF 400 mg and DCV 60 mg (60.6%), or SOF 400 mg and DCV 90 mg (39.4%) (Table 1). At baseline, 16 participants (48.5%) were undergoing methadone therapy, and 7 (21.2%) had a urinary positive test for methamphetamine. Compared with HCV-seronegative PWID, PWID living with HCV were more frequently co-infected with HIV (42.4% vs 13.3%). The characteristics of the population analyzed were comparable to those of the initial population (Table S1). In addition, baseline characteristics were broadly comparable between included and excluded participants according to sequencing success (Table S2).

**TABLE 1:** Baseline characteristics of HCV-positive and HCV-seronegative PWID^*e*^

	HCV-seronegative PWID (*n* = 30)	HCV-positive PWID (*n* = 33)	*P* value
Socio-demographic data			
Gender, male/transgender	27 (90.0)	30 (90.9)	1^*b*^
Age (in years), median (IQR)	40 (34; 47)	43 (38; 51)	0.12^*d*^
Viral infections; *n* (%)			
HIV coinfection	4 (13.3)	14 (42.4)	0.01^*c*^
Current HBV infection	N.A.	0 (0)	N.A.
Treatment; *n* (%)			
Declare being on methadone- assisted therapy	16 (53.3)	16 (48.5)	0.70^*c*^
DAA treatment			N.A.
SOF400/DCV60	N.A.	20 (60.6)	
SOF400/DCV90	N.A.	13 (39.4)	
Receiving ARV treatment (among HIV-infected participants)	4 (100)	10 (76.9)^*a*^	0.54^*b*^
Drug consumption; *n* (%)			
Heroin			
Number of years of injection			0.04^*b*^
Less than 5 years	2 (6.7)	0 (0.0)	
5–10 years	7 (23.3)	8 (24.2)	
10–15 years	15 (50.0)	9 (27.3)	
Over 15 years	6 (20.0)	16 (48.5)	
Frequency of injections per month			0.29^*c*^
Less than once a day	12 (40.0)	9 (27.3)	
Daily	18 (60.0)	24 (72.7)	
Methamphetamine			
Positive urinary test	9 (30.0)	7 (21.2)	0.42^*c*^

^*a*^ One missing value.

^*b*^ Fisher’s exact test.

^*c*^ Chi-squared test.

^*d*^ Wilcoxon rank test.

^*e*^ ARV, antiretroviral; DAA, direct-acting antivirals; DCV, daclatasvir; IQR, interquartile range; N.A., not applicable (HBV status not available for HCV-seronegative PWID); SOF, sofosbuvir.

### mtDNA deletome

After applying quality control procedures described in Materials and Methods, significant mtDNA deletions were detected in a cross-sectional observation, in three PWID living with HCV (9.1%) at baseline, while none were observed in the HCV-seronegative group (Table 2). The heteroplasmy rate, defined as the proportion of deleted mtDNA relative to the wild-type form, ranged from 0.10% up to 0.70%, with a cumulative median of 0.22%. No significant associations were found between mtDNA deletion and gender, age, HIV co-infection, receiving ARV treatment, or drug consumption (Table S3).

**TABLE 2:** Baseline mtDNA deletion profiles in HCV-positive and HCV-seronegative PWID^*a*^

	HCV-seronegative PWID (*n* = 30)	HCV-positive PWID (*n* = 33)
PWID with mtDNA alteration; *n* (%)	0 (0)	3 (9.1)
Deletion events; *n*	0	3
Cumulative heteroplasmy; median % (IQR)	0 (0.00–0.00)	0.22 (0.10–0.70)
PWID with heteroplasmy > 1%; *n* (%)	0 (0)	0 (0)

^*a*^ IQR, interquartile range; mtDNA, mitochondrial DNA.

Looking at the longitudinal case series of mtDNA alterations among DAA-treated PWID, only 26 PWID out of 33 were analyzed with a complete set of sequencing data. The prevalence of mtDNA deletions increased over time from 7.7% at baseline to 19.2% at W12, remaining stable at W48 (Table 3). Although the total number of mtDNA deletion events rose from 2 (baseline) to 8 (W48), the cumulative heteroplasmy level declined over time, from 0.40% at baseline to 0.09% at W48, and no participant had a heteroplasmy rate exceeding 1%.

**TABLE 3:** Longitudinal evolution of mtDNA deletions during DAA treatment in PWID living with HCV^*a*^

	Baseline (*n* = 26)	W12 (*n* = 26)	W48 (*n* = 26)
PWID with mtDNA alteration; *n* (%)	2 (7.7)	5 (19.2)	5 (19.2)
Deletion events; *n*	2	7	8
Cumulative heteroplasmy; median % (IQR)	0.40 (0.10–0.70)	0.15 (0.07–0.36)	0.09 (0.06–0.17)
PWID with heteroplasmy > 1%; *n* (%)	0 (0)	0 (0)	0 (0)

^*a*^ IQR, interquartile range; mtDNA, mitochondrial DNA.

No significant associations were found between mtDNA deletions and clinical outcomes, including hepatic or splenic complications or changes in liver stiffness assessed 3 years after study completion.

### mtDNA point variants

**Fig 2:**
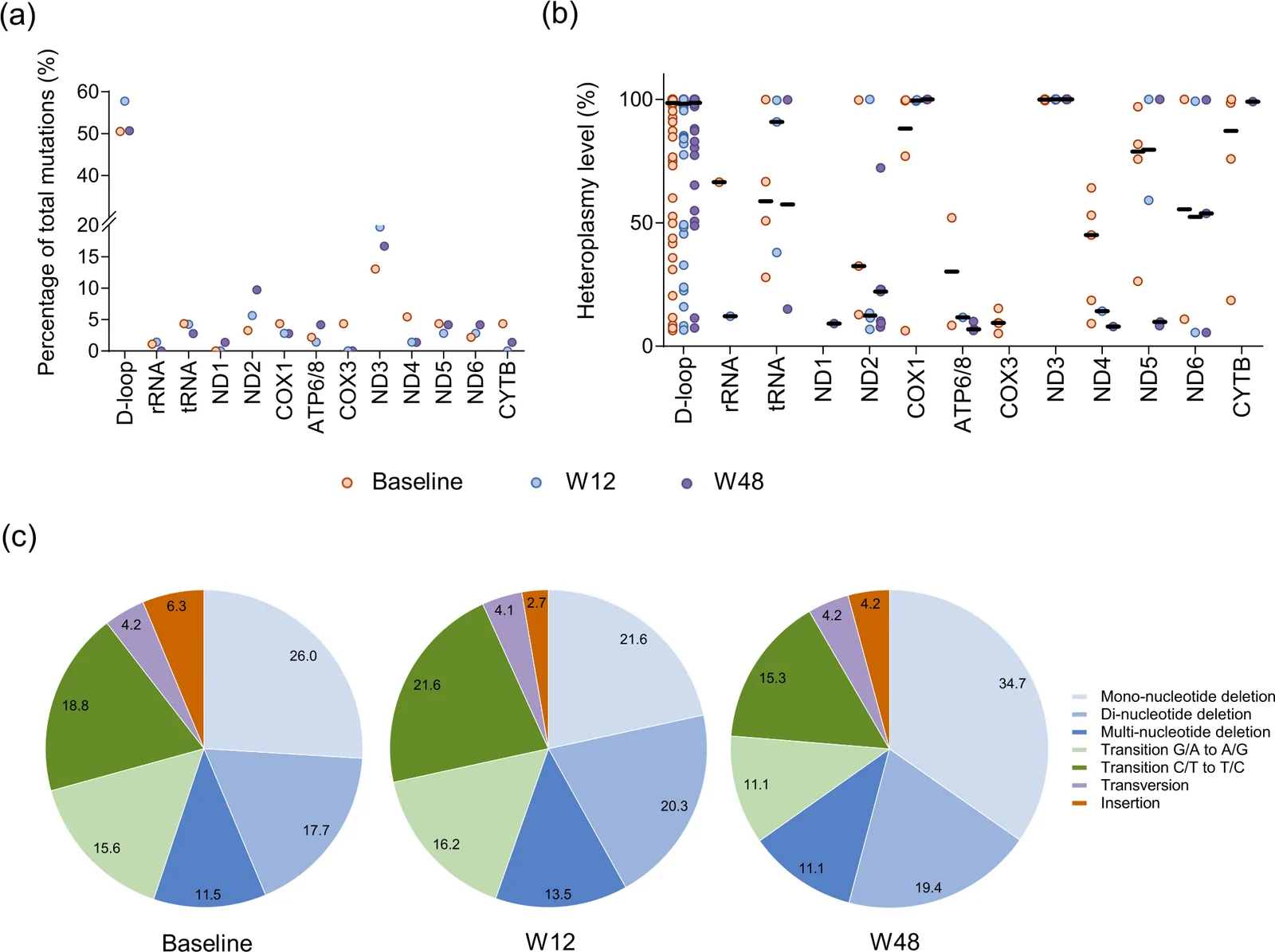
Longitudinal dynamics of mtDNA variants in PWID living with HCV during DAA treatment. (**a**) Genomic distribution and (**b**) heteroplasmy levels of mtDNA variants at baseline, W12, and W48. (**c**) Evolution of variant type profiles over time, expressed as percentages (*n* = 96 at baseline; *n* = 74 at W12; and *n* = 72 at W48).

Variants were defined as high-confidence single-nucleotide variants detected by both variant callers, without strand bias, and excluding haplogroup-defining polymorphisms. mtDNA variants were detected in 30/33 (90.9%) PWID living with HCV and 29/30 (96.7%) HCV-seronegative participants at baseline. The incidence and genomic distribution of mtDNA variants were similar between groups (*P* = 0.86) (Fig. 1a). Variants were most frequently located in the D-loop region, a non-coding region, accounting for 54.8% of variants in PWID living with HCV and 53.2% in HCV-seronegative PWID. Overall heteroplasmy levels did not differ significantly between groups (*P* = 0.83) (Fig. 1b). The distribution of variant types was also comparable (*P* = 0.99) (Fig. 1c). Transitions were the most frequent substitutions (38.1% for HCV-positive vs 39.0% for HCV-seronegative participants), followed by single-nucleotide deletions (23.0% for HCV-positive vs 22.9% for HCV-seronegative participants). Recurrent single-nucleotide deletions were found at positions 247, 513, and 8270 within the D-loop region, and recurrent point variants at positions 10398 and 10400 in the *ND3* gene (Fig. 1d). The m.10398A>G alteration was the only non-synonymous variant identified, resulting in an amino acid substitution, and was observed in 16.7% of HCV-seronegative PWID and 24.2% of HCV-positive PWID. Notably, all variants identified were previously reported as common polymorphisms in population databases (MITOMAP, gnomAD, and Helix; frequency > 0.1%), supporting their likely non-pathogenic nature and indicating that they mainly reflect individual mitochondrial genetic backgrounds. Longitudinal analysis revealed modest changes in the distribution of mtDNA variants across the genome (Fig. 2a). Although not statistically significant (*P* = 0.49), a trend toward increased variants in coding genes such as *ND2* was observed at W48. Heteroplasmy levels of these variants remained generally stable over time (*P* = 0.36), with no significant differences across the three time points (baseline, W12, and W48) for any individual region (Fig. 2b). The overall distribution of variant types was stable between baseline, W12, and W48 (*P* = 0.94) (Fig. 2c). At W48, a relative increase in single-nucleotide deletions was observed, representing 34.7% of all variants compared to 26.0% at baseline and 21.6% at W12, although this difference was not statistically significant.

In PWID living with HCV, low-heteroplasmy treatment-emergent variants were detected in only one participant (3.8%) at W12 but were not found at W48. One variant was located in the gene *MT-ND1* (m.663A>G); the others were located at positions 8794 in *MT-ATP6* and 11084 in *MT-ND4* (Table S4). These variants were reported as polymorphisms in population databases (MITOMAP, gnomAD, and Helix; frequency > 0.1%), supporting their likely non-pathogenic nature; therefore, they were not further investigated in this study.

## DISCUSSION

Low-level mtDNA instability, reflected mainly by large-scale deletions and rare transient point variants, was detected both at baseline in PWID living with HCV and during follow-up after DAA initiation in blood leukocytes. None of these alterations reached a heteroplasmy rate of 1% at the end of treatment or after 9 months. These low-level alterations in blood leukocytes were not associated with detectable short-term clinical correlates in our cohort. However, this low level may underestimate mitochondrial genomic burden in post-mitotic tissues, precluding conclusions regarding longer-term, tissue-specific effects.

Altogether, these findings support the hypothesis that HCV infection may contribute to mtDNA instability and confirm previous studies which used a double quantitative PCR approach in the same cohort (27). Consistently, our current data show an increase in mtDNA deletion during and after DAA treatment, reaching 19.2% at both follow-up visits. Interestingly, these deletions remained at low-heteroplasmy levels, since no participants exhibited deletions exceeding the 1% heteroplasmy level. This suggests limited clonal expansion or effective compensatory mechanisms.

mtDNA variants were more tricky to classify as they could result from adaptation to the pathophysiological situation and be amplified if they confer a selective advantage, sometimes with pathogenic effects. The high baseline burden likely reflects chronic exposure to mitotoxic agents, such as heroin, methamphetamine, and antiretroviral drugs. Most variants occurred in the D-loop, a non-coding regulatory region of mtDNA with high mutational tolerance, and a large proportion were synonymous, consistent with findings in ART-treated individuals living with HIV (17, 25, 31). The only recurrent non-synonymous variant detected in both groups was the m.10398A>G variant in the *ND3* gene, a common polymorphism associated with altered mitochondrial efficiency and bioenergetic effects (13, 32). This variant has been linked to tumorigenesis in breast and prostate cancer among African–American populations and has also been reported in association with brain tumors (13, 33). Nonetheless, because m.10398A>G is widely distributed among several mitochondrial haplogroups, its functional and clinical significance remains ambiguous (13).

Several studies have reported an association between ART and mtDNA point mutations in ART-treated individuals living with HIV (16, 17, 31, 34, 35). An accumulation of mtDNA mutations during ART treatment, with an increased heteroplasmy level in specific regions, has been described in some studies (16, 17), whereas others reported no accumulation (36–38). In our cohort, PWID living with HCV were frequently co-infected with HIV and exposed to long-term ART, both previously associated with mitochondrial instability (22). However, most participants living with HIV had sustained virological suppression, suggesting a relatively stable chronic exposure background with potential mitochondrial adaptation over time. Finally, DAA treatment was not associated with an increase in mtDNA variants, and heteroplasmy levels remained generally stable over time despite some fluctuations. Discrepancies between studies may reflect differences in drug class, treatment duration, virological control, population susceptibility, and methodological approaches. The few treatment-emergent variants identified at W12 were detected at low-heteroplasmy levels in a single participant and were no longer observed at W48. In addition, these variants corresponded to previously reported polymorphisms in population databases, supporting their likely non-pathogenic nature. Therefore, our findings do not support the persistence or accumulation of clinically relevant mtDNA point variants following DAA exposure in this cohort.

Our study has several limitations. First, amplification failures for some long-range PCR fragments reduced the number of samples available for complete mtDNA analyses at specific time points. The relatively small sample size may have limited the statistical power to detect multivariate associations or subtle longitudinal effects. The monocentric design may limit the applicability of our findings to broader populations. Furthermore, participants were exposed to other mitotoxic factors, including illicit drugs, HIV infection, and antiretroviral therapies. These exposures likely represent long-term background factors rather than acute or time-varying covariates during follow-up, thereby limiting the ability to assess their potential contribution as time-varying determinants of mtDNA alterations. Additionally, the choice of blood as the analyzed tissue, characterized by high cell renewal, may underestimate the extent of mtDNA alterations compared to post-mitotic tissues such as brain, heart, and muscle (39). Rapid cell renewal may dilute the detection of persistent or cumulative mutations, thereby masking subtle but potentially relevant genomic instability.

In conclusion, we detected low levels of mtDNA instability in blood leukocytes among PWID receiving DAA treatment. These findings provide reassuring, although preliminary, evidence regarding the short-term mitochondrial safety of DAA regimens. This observation could lead to additional investigations on more vulnerable organs, such as the slow-cycling ones, as well as long-term mitochondrial surveillance.

## Data Availability

The data presented in this study are available on request from the corresponding author.

## SUPPLEMENTAL MATERIAL

Supplemental tables spectrum.01946-26-s0001.docx Tables S1 to S4.

ASM does not own the copyrights to Supplemental Material that may be linked to, or accessed through, an article. The authors have granted ASM a non-exclusive, world-wide license to publish the Supplemental Material files. Please contact the corresponding author directly for reuse.
