# Supplementary material for: Low-level of mitochondrial DNA alterations during direct-acting antiviral treatment in leukocytes of people who inject drugs living with HCV
Source: Microbiol Spectr. 2026 Oct 9;14(11):e01946-26. doi: 10.1128/spectrum.01946-26 (PMC13729135; doi:10.1128/spectrum.01946-26)
Supplement: Supplementary file 1 [file spectrum.01946-26-s0001.docx]

**Supplementary tables**

**Supplementary table 1.** Baseline characteristics of PWID according to the selection in the sample

|  | **PWID not selected**  **(n = 1,381)** | **PWID analysed**  **(n = 63)** | **p-value** |
| --- | --- | --- | --- |
| **Socio-demographic data** |  |  |  |
| **Gender**, Male/Transgender | 1,310 (94.9)^-^ | 57 (90.5) | 0.14* |
| **Age** (in years); median [IQR] | 41 [35; 47]^-^ | 42 [37; 49] | 0.28^$^ |
| **Viral infections**; n (%) |  |  |  |
| HIV coinfection | 377 (27.3)^-^ | 18 (28.6) | 0.83^+^ |
| **Treatment**; n (%) |  |  |  |
| Declare being on methadone-assisted therapy | 565 (41.1)^§^ | 32 (50.8) | 0.13^+^ |
| Receiving ARV treatment  (among HIV-infected participants) | 313 (91.8)^£^ | 14 (82.4)^-^ | 0.17* |
| **Drug consumption**; n (%) |  |  |  |
| **Heroin** |  |  |  |
| Number of years of injection |  |  | 0.09^+^ |
| Less than 5 years  5 to 10 years  10 to 15 years  Over 15 years | 146 (10.6)^-^  384 (27.8)  369 (26.7)  481 (34.9) | 2 (3.2)  15 (23.8)  24 (38.1)  22 (34.9) |  |
| Frequency of injections per month |  |  | 0.51^+^ |
| Less than once a day  Daily | 407 (29.5)^-^  973 (70.5) | 21 (33.3)  42 (66.7) |  |
| **Methamphetamine** |  |  |  |
| Positive urinary test | 466 (33.7) | 16 (25.4) | 0.17^+^ |

**^+^** Chi-squared test; * Fisher exact test; ^$^ Wilcoxon rank test

^-^ One missing value; ^§^ 6 missing values; ^£^ 36 missing values

Abbreviations: ARV, antiretroviral; IQR, interquartile

|  | **HCV-seronegative**  **PWID without DNA**  **(n = 8)** | **HCV-seronegative**  **PWID with DNA**  **(n = 30)** | **p-value** | **HCV-positive**  **PWID without DNA**  **(n = 18)** | **HCV-positive**  **PWID with DNA**  **(n = 33)** | **p-value** |
| --- | --- | --- | --- | --- | --- | --- |
| **Socio-demographic data** |  |  |  |  |  |  |
| **Gender**, Male/Transgender | 7 (87.5) | 27 (90.0) | 1* | 17 (94.4) | 30 (90.9) | 1* |
| **Age** (in years); median [IQR] | 46 [35; 53] | 40 [34; 47] | 0.35^$^ | 43 [39; 45] | 43 [38; 51] | 0.61^$^ |
| **Viral infections**; n (%) |  |  |  |  |  |  |
| HIV coinfection | 1 (12.5) | 4 (13.3) | 0.95**^+^** | 12 (66.7) | 14 (42.4) | 0.10^+^ |
| **Treatment**; n (%) |  |  |  |  |  |  |
| Declare being on methadone-assisted therapy | 4 (100)^ϕ^ | 16 (84.2)^€^ | 0.39**^+^** | 9 (60.0)^¥^ | 16 (59.3)^§^ | 0.96^+^ |
| Receiving ARV treatment  (among HIV-infected participants) | 1 (100) | 4 (100) | - | 12 (100) | 10 (76.9)^-^ | 0.22* |
| **Drug consumption**; n (%) |  |  |  |  |  |  |
| **Heroin** |  |  |  |  |  |  |
| Number of years of injection |  |  | 0.09^+^ |  |  | 0.78^+^ |
| Less than 10 years  Over 10 years | 5 (62.5)  3 (37.5) | 9 (30.0)  21 (70.0) |  | 5 (27.8)  13 (72.2) | 8 (24.2)  25 (75.8) |  |
| Frequency of injections per month |  |  | 0.15^+^ |  |  | 0.65^+^ |
| Less than once a day  Daily | 1 (12.5)  7 (87.5) | 12 (40.0)  18 (60.0) |  | 6 (33.3)  12 (66.7) | 9 (27.3)  24 (72.7) |  |
| **Methamphetamine** |  |  |  |  |  |  |
| Positive urinary test | 6 (75.0) | 9 (30.0) | 0.02**^+^** | 5 (27.8) | 7 (21.2) | 0.60^+^ |

**Supplementary table 2.** Baseline characteristics of participants with and without DNA amplification by HCV status

**^+^** Chi-squared test; * Fisher exact test; ^$^ Wilcoxon rank test

^-^ One missing value; ^¥^ 3 missing values; ^ϕ^ 4 missing values; ^§^ 6 missing values; ^€^ 11 missing values

**Supplementary table 3.** Determinants of mtDNA deletions in PWID living with HCV (n = 33)

|  | **mtDNA deletion** | | **p-value** |
| --- | --- | --- | --- |
|  | **negative**  **(n = 30)** | **positive**  **(n = 3)** |  |
| **Gender**, Male/Transgender | 27 (90.0) | 3 (100.0) | 1* |
| **Age** (in years); median [IQR] | 43 [38; 51] | 48 [38; 54] | 0.062^$^ |
| **HIV coinfection**; n (%) | 12 (40.0) | 2 (66.7) | 0.56* |
| **Receiving ARV treatment**  (among HIV-infected participants); n (%) | 8 (72.7)^-^ | 2 (100) | 1* |
| **Drug consumption**; n (%) |  |  |  |
| Daily heroin injection during the last month  Positive methamphetamine urinary test | 22 (73.3)  7 (23.3) | 2 (66.7)  0 (0.0) | 1*  1* |

* Fisher exact test; ^$^ Wilcoxon rank test

^-^ One missing value

Abbreviations: ARV, antiretroviral; IQR, interquartile; mtDNA, mitochondrial DNA

**Supplementary table 4.** Description of transient treatment-emergent variants in PWID living with HCV (n = 26)

| **Gene** | **Position** | **Ref** | **Alt** | **Amino-acid change** | **Heteroplasmy level (%)** |
| --- | --- | --- | --- | --- | --- |
| rRNA | 663 | A | G | N.A. | 12.2 |
| ATP6 | 8794 | C | T | H90Y | 11.7 |
| ND4 | 11084 | A | G | T109A | 14.2 |

Abbreviations: Ref = reference allele; Alt = alternative allele
